# Zonulin and copeptin relation to some metabolic markers in school-aged obese children

**DOI:** 10.1186/s12887-024-04617-1

**Published:** 2024-02-24

**Authors:** Sahar Abd El-Raufe El-Masry, Rehab A. Mahmoud, Nayera E Hassan, Manal M. Aly, Hanaa Reyad Abdallah, Sherin Hamdy, Safinaz Megahed, Dina Y. Elalfy

**Affiliations:** 1 Biological Anthropology Department, Medical Research and Clinical Studies Institute, National Research Centre, 33 El-Buhouth St., Dokki, Giza, 12622 Egypt; 2 Faculty of Post Graduate Childhood Studies, Ain-Shams University, Cairo, Egypt; 3 Clinical and Chemical Pathology Department, Medical Research and Clinical Studies Institute, National Research Centre, Giza, Egypt

**Keywords:** Zonulin, Copeptin, Fat distribution, Body composition, Metabolic markers, Egyptian obese children

## Abstract

**Background:**

Using Zonulin and Copeptin as potential obesity markers in children, hasn’t yet been focused.

**Aim:**

To evaluate the association between serum levels of both Zonulin and Copeptin with the obesity markers, and to assess their role as metabolic disturbance predictors in obese children.

**Methods:**

A case-control study comprised 111 Egyptian children (45 males and 66 females); aged 6–10 years to avoid the effect of puberty (prepubertal). They were classified according to their body mass index (BMI) percentiles into: 72 obese (BMI ≥ 95th ), and 39 control ones (BMI > 15th - <85th ), based on the Egyptian Growth Charts for children and adolescents. Anthropometric parameters and blood pressure were measured, and body composition analysis, lipid profile, Zonulin, and Copeptin levels were assessed.

**Results:**

The obese group showed a significantly higher value of Copeptin and a lower value of Zonulin than the control one Also, the obese group showed significant negative correlations between Zonulin and both anthropometric obesity markers and body composition, whereas Copeptin showed significant positive ones. Moreover, significant positive correlations were found between Copeptin and both body weight and fat distribution. Insignificant correlations were observed between both serum Zonulin and Copeptin levels and blood pressure and lipid profile.

**Conclusion:**

Zonulin and Copeptin cannot be used as metabolic disturbance predictors, among Egyptian children, as they were insignificantly correlated with lipid profile or blood pressure.

## Introduction

Overweight and obesity are regarded as essential causes of morbidity and mortality worldwide. Their prevalence has dramatically increased and has become a global pandemic issue, among both children and adolescents [1]. This global problem imposes the discovery of novel obesity biomarkers, as well as, its associated metabolic disorders [2]. In Egypt, *Hassan et al.* [3]; in 2018; reported that the estimated prevalence of overweight and obese Egyptian children aged 6 to 18 years in Giza Governorate, was 11.0% and 19.5%, respectively based on the Standardized Egyptian Growth Curves for children and adolescents [4]. Whereas it was 13.3% and 10.5%, respectively among Upper Egypt primary schools’ children, according to the Centers for Disease Control “CDC” growth chart, as reported by *Abd El-Aty et al.;* in 2020 [5].

Obesity can be attributed to several genetic, environmental and behavioral factors [6]. *Tuncay et al.* [7], suggested that the pro-inflammatory peptides released due to intestinal barrier dysfunction, in obesity, may play an important role. Moreover, serum zonulin level may be regarded as a valuable marker of intestinal permeability [8].

Zonulin, is a protein that acts via modulating the intracellular tight junctions, thus increasing the intestinal permeability in the small intestine. The increased exposure to pathogens and allergens, due to weakening of the intestinal barrier leads to obesity and metabolic abnormalities [9]. The intestinal epithelium plays a major role in nutrient digestion, solutes and electrolytes absorption in addition to acting as a barrier; tightly controlling antigen leakage from the intestinal lumen to the sub-mucosa. In turn, this leakage disturbs the balance between immune response and tolerance, and hence causes inflammation. Secondary to increased Zonulin, the only known physiological modulator of intercellular tight junctions is lost, and hence allows uncontrolled attack of microbial and dietary antigens [10]. Little information is available about the relationship between Zonulin and obesity, and the possibility of its use as valuable marker for obesity and metabolic disorders in children and adolescents [11].

Copeptin, the Arginine vasopressin (AVP) surrogate, is a peptide co-synthesized together with the AVP hormone in the endothelial cells and pituitary gland. It is characterized by its vasoactive, immune modulating, and metabolic properties, short half-life, higher stability, being easier to measure than the active hormone AVP and requiring fewer pre-analytical procedures. More recently, Copeptin has been used as a reliable marker of serum AVP concentration; as it is secreted in equimolar amounts with AVP [12–14].

Although, several studies conducted on adult populations have assessed the efficacy of Copeptin in diagnosing pathways of AVP-related diseases [14, 15], but in children no studies were found except that of *Tuli et al.* [2] who studied the plasma level of Copeptin and its use as a significant diagnostic tool for AVP-related diseases in children .

Reviewing the literature, no studies were found to assess the relationship between Zonulin and Copeptin as potential markers for obesity and their associated disorders in both children and adolescents. Hence, the aim of the current study was to assess the association between both Zonulin and Copeptin serum levels and the obesity and metabolic markers, in addition to their roles as potential predictor markers for metabolic disturbances in obese children.

## Subjects and methods

### Subjects

This case-control study comprised 111 children (45 ♂ and 66 ♀); whose ages ranged between 6 and 10 years (to avoid the effect of puberty) i.e. pre-pubertal. The exclusion criteria (by full History taking and clinical examination) were the presence of any sign of puberty according to Tanner stage, presence of identified causes of obesity (genetic syndromes, chromosomal or endocrinal disorders), chronic diseases (cardiovascular, gastrointestinal, and respiratory), or drug use like steroids; that would interfere with the type of obesity and affect the normal growth of the children. Also, any child with a BMI between 85th to 95th percentiles (overweight) was excluded from the study. All participating obese children were suffering from exogenous simple obesity. Based on Egyptian Growth Curves for children and adolescents, BMI ≥ 95th percentile was considered obese, for age and sex [4]. According to their BMI percentiles, participants were classified into 2 groups: group (1) comprising 72 obese children (BMI ≥95th percentile), and group (2) comprising 39 control ones (BMI >15th - <85th percentiles). The study was conducted; during the period between December 2018 and February 2021; on children who attending the “Management of Visceral Obesity and Growth Disturbances clinic”, in the **“MRCE”** (the Medical Research Centre of Excellence) of the **“NRC” the** National Research Centre, Giza, Egypt.

#### Ethical consent

Approvals from Ethics Committees of both Faculty of Postgraduate Childhood Studies and the “National Research Centre” were obtained (Approval No. 17/123). After explaining the promising benefits of the study in ascertaining the impact of obesity on health, informed written consent was obtained from either parent.

## Methods

For each child the following was done: blood pressure assessment, anthropometric measurements, and body composition and laboratory investigations.

### Blood pressure assessment

A standardized mercury sphygmomanometer was used to measure both systolic and diastolic blood pressures; with the subject in sitting position and with an appropriate cuff covering at least 2/3 of the left upper arm length, and not surpassing on the antecubital space. Three consecutive readings were taken, then if the error was acceptable, the mean was recorded [16].

### Anthropometric measurements

The anthropometric parameters measured included: weight (Wt), height (Ht), waist circumference (WC), hip circumference (HC) and the skin folds thicknesses of: triceps, biceps, sub scapular, suprailiac and abdominal, following the “IBP” recommendations [17].

Body weight was recorded using a standing digital SECA scale balance (Model 707), to the nearest 0.1 kg. Average body height was recorded to the nearest 0.1 cm, after being measured three successive times, using a wall mounted Holtain Stadiometer. Waist circumference (WC) was measured using simple non-stretchable plastic measuring tape, to the nearest 0.1 cm, at a level midway between the lower rib margin and iliac crest, all around the body in horizontal position. Hip circumference (HC) was measured using simple non-stretchable plastic measuring tape, measuring the largest diameter at the maximum extension of the buttocks above the symphisis pubis overlapping the apex of the buttocks, to the nearest 0.1 cm. Skin fold thickness was measured to the nearest 0.1 mm., at the left side of the body; with the Holtain Skin fold Calipers [Holtain ltd., UK, Wales, Cross well, no. 646,420].

From the previous measurements, the following ratios and indices were calculated: body mass index (BMI); using the formula: BMI = Weight (Kg) / [Height (m^2^)] [18]; waist/hip ratio in cm (WHR), waist/height ratio in cm. (WHTR), peripheral obesity index (= triceps + biceps skin fold thickness) in mm. and central obesity index (= Sub scapular + Suprailiac + abdominal skin fold thicknesses) in mm.

After calculating the BMI for every child, it was plotted, according to his age, on the Egyptian Sex-Specific Growth Chart [4], as a result they were classified as 72 obese and 39 normal weight (control) groups.

### Body composition analysis

Body Composition Analysis was measured using computerized Holtain’s BCA (Holtain Body Composition Analyzer (Holtain ltd., UK, Wales, Crosswell No. 646512). Impedance measurement was done 2 or 3 hours after eating and within 30 minutes of voiding urine. Being in supine position, the child laid with the arms at the side of his body, but not touching it. Following the manual instructions, four self-adhesive disposable electrodes were applied; 2 to the back of the right wrist joint and 2 to the front of the right ankle joint. A constant current of 800 µA at 50 kHz was applied across the body. The impedance of the body under test, according to Ohm’s Law, was given by the corresponding voltage drop. The subject’s sex, age, weight and height; approximated to the nearest unit; together with the measured value of the impedance were fed into specially programmed ‘organizer’ which then displays the BF% (the fraction of the total body fat mass), Fat Mass (FM); the fraction of the total body weight that is adipose tissue), Fat-Free Mass (FFM); the fraction of the total body weight that is not adipose tissue), body water content (liter), Basal Metabolic Rate (BMR) in kilo calories at rest at rest: the rate at which the body uses energy, to maintain vital functions.

### Laboratory investigations

A 12 h of fasting, a 5 ml venous blood sample of was obtained from every child. Samples were left to clot, then centrifuged and sera were separated to be kept at -80 °C for further assessment of lipid profile, zonulin and Copeptin levels.

Quantitative enzymatic colorimetric determination of serum triglycerides was assessed using test kit code no: SU033, SU034, SU035 (CHEMELEX, S.A., Barcelona). Assessment of total cholesterol was done using, CHOD-POD enzymatic colorimetric method, serum test kit Ref: 101–0440/101–0526 (CHRONOLAB SYSTEMS, Barcelona). HDL was assessed using kit code no: SU014 (CHEMELEX, S.A., Barcelona). Serum triglycerides, total cholesterol and HDL were assessed according to the method of *Tietz* [19]. While LDL was assessed using kit REF: 99 06 10 (QUIMICA CLINICA APLICADA S.A., Spain) according to Polvinyl Sulphate method of *Demacker et al.* [20].

Both serum Zonulin and Copeptin were assessed using BRAHMSCT-proAVP LIA Kit (B.R.A.H.M.S. GmbH, Hennigsdorf Germany). Enzyme Linked Immunosorbent Assay (ELISA) was used to assess Serum Zonulin, based on the principle of competitive enzyme immunoassay. In this assay, the micro plate in the kit is pre-coated with antibody specific to the Zonulin. The standard is reconstituted and prepared by serial dilution with sample diluents. The serum samples are diluted 1:2000 for zonulin. Standards and samples are loaded into the appropriate micro titer plate wells and any Zonulin present is bound by immobilized antibody. A standard curve of known concentration of analyte is established and the concentration of Zonulin in the samples is calculated accordingly. The sensitivity of ELISA assays for Zonulin is 0.156 ng/mL; with a detection range of 0.6–10.8 ng/ mL. Serum Copeptin samples were collected and then assessed; in tubes containing (EDTA); in parallel to serum osmolality, by an immune-luminometric assay, with detection limit < 0.4 pmol/L and functional assay sensitivity < 1 pmol/L, a detection range 15–270 pg/ L.

### Statistical analysis

The gathered data were statistically analyzed using the SPSS computer program version 16 (Statistical Package for Social Science). Data Normality test was done using the Kolmogorov-Smirnov. Descriptive statistics (mean ± SD) were calculated for the anthropometric and body composition parameters and laboratory investigations. Quantitative parametric comparison of the 2 groups was done using the Mann-Whitney test, to find group differences. To evaluate the correlations between both Zonulin and Copeptin and all the studied variables, Spearman´s correlation was used. In all statistical analyses, the value of *P* < 0.01 was regarded as highly significant, while *P* < 0.05 was considered statistically significant.

## Results

Non-parametric tests were carried out since the studied variables were not normally distributed (i.e. BMI, WC, HC, all skin fold thicknesses, body composition and laboratory investigations; particularly Zonulin and Copeptin). Moreover, insignificant sex differences were reported among all the studied variables (Table 1), hence the analyses were done without sex differentiation.

Table 2 revealed highly significant differences between obese and control groups (blood pressure, anthropometric and body composition parameters), where the obese group had higher values; except BMR where the control group had higher values. Concerning the laboratory investigations, the obese group had higher significant values of total cholesterol (*p* = 0.030) and lower highly significant values of HDL (*p* = 0.000). Levels of both LDL and TG showed insignificant differences between the obese and control group. The obese group had a higher significant value of Copeptin (*p* = 0.031) and a lower significant value of Zonulin (*p* = 0.029).

Table 3 presented Spearman’s correlation analysis, between both Zonulin and Copeptin and the other studied parameters, among the obese children. Serum Zonulin showed significant negative correlation, whereas serum Copeptin showed significant positive correlations with the obesity markers: [BMI (*p* = 0.001, *p* = 0.000), WC (*p* = 0.006, *p* = 0.001), HC (*p* = 0.000), WHTR (*p* = 0.006, *p* = 0.002), SCSF (*p* = 0.002, *p* = 0.006)] and body composition [FM (*p* = 0.035, *p* = 0.009), FFM (*p* = 0.004, *p* = 0.000), TBW (*p* = 0.011, *p* = 0.001), and BMR (*p* = 0.006, *p* = 0.001)]. Moreover, serum Copeptin had significant positive correlation with Weight (*p* = 0.017), and fat distribution [TSF (*p* = 0.032), ABSF (*p* = 0.004), and Central obesity index (*p* = 0.007)]. Moreover, serum Zonulin and Copeptin had significant negative correlation with each other (*P* = 0.000). Results of the present study revealed insignificant correlations between both Zonulin and Copeptin and all of the studied lipid profile and blood pressure.

Table 4 showed Spearman’s correlation analysis between both Zonulin and Copeptin and the other studied variables among control group. It revealed that serum Zonulin had significant negative correlation, while serum Copeptin had significant positive correlations with Weight (*p* = 0.000), Height (*p* = 0.000), HC (*p* = 0.031, *p* = 0.007), and TG (*p* = 0.029, *p* = 0.011). Whereas, Serum Zonulin had significant positive correlation with WHTR (*p* = 0.001, *p* = 0.000), and HDL (*p* = 0.012).While, serum Copeptin had significant negative correlation with some obesity markers [WHR (*p* = 0.035), WHTR (*p* = 0.000)], fat distribution [most of the investigated skinfold thickness: BSF (*p* = 0.004), TSF (*p* = 0.018), SCSF (*p* = 0.021), ABSF (*p* = 0.004) and peripheral obesity index (*p* = 0.004)]. Although, the findings of the present study revealed highly significant negative correlations between Zonulin and Copeptin (*P* = 0.000), yet both Zonulin and Copeptin showed insignificant correlations with blood pressure (SBP and DBP), cholesterol and LDL.

**Table 1:** Sex differences in investigated clinical, anthropometric, body composition and laboratory parameters among total sample (Mann-Whitney test)

Parameter	Male (*n* = 45)		Female (*n* = 66)		z	p
	Mean	SD	Mean	SD		
Blood pressure						
SBP (mm Hg)	102.15	18.88	103.11	11.36	-0.387	0.699
DBP (mm Hg)	63.44	12.10	63.56	7.68	-0.974	0.330
Anthropometry						
Weight (Kg)	46.30	18.36	45.96	20.57	-0.003	0.998
Height (cm)	133.91	14.48	133.14	18.05	-0.051	0.959
BMI (kg/m2)	24.70	6.36	24.40	7.27	-0.063	0.950
WC (cm)	79.24	16.66	78.83	18.66	-0.123	0.902
HC (cm)	87.13	17.11	87.92	22.26	-0.183	0.855
WHR (cm/cm)	0.91	0.07	0.90	0.08	-0.277	0.782
WHTR (cm/cm)	0.59	0.10	0.59	0.10	-0.228	0.819
Skin fold thickness						
BSF (mm)	16.52	8.20	15.48	8.00	-0.535	0.593
TSF (mm)	23.55	10.65	22.84	11.35	-0.355	0.723
SCSF (mm)	24.52	14.05	24.90	16.11	-0.206	0.836
SISF (mm)	22.81	13.92	20.94	13.28	-0.629	0.529
ABSF (mm)	24.48	14.96	24.38	15.67	-0.026	0.979
Peripheral obesity (mm)	40.07	18.60	38.32	18.91	-0.403	0.687
Central obesity (mm)	71.81	40.51	70.22	42.01	-0.049	0.961
Body composition						
TBW (Kg)	41.41	25.45	42.15	25.08	-0.288	0.774
FFM (Kg)	27.62	9.72	27.10	9.96	-0.357	0.721
FM (Kg)	17.44	11.50	17.63	14.67	-0.484	0.629
BF%	31.93	18.15	27.15	28.84	-0.007	0.995
BMR (Kcal)	2393.56	920.55	2426.84	933.54	-0.424	0.671
Laboratory parameters						
Lipid profile						
Chol (mg/dl)	165.82	36.48	166.62	26.05	-0.828	0.408
TG (mg/dl)	88.51	29.11	86.00	27.40	-1.320	0.187
HDL (mg/dl)	50.27	25.11	48.58	13.69	-0.721	0.471
LDL (mg/dl)	66.84	10.47	66.86	8.24	-0.172	0.864
Zonulin (ng/mL)	0.96	1.07	1.22	1.63	-0.274	0.784
Copeptin (pmol/l)	198.67	109.92	203.12	110.41	-0.172	0.863

SBP: Systolic blood pressure, DBP: Diastolic blood pressure, BMI: Body Mass Index, WC: Waist circumference, HC: Hip circumference, WHR: Waist/Hip ratio, WHTR: Waist/Height ratio, BSF: Biceps Skin Fold, TSF: Triceps Skin Fold, SCSF: Subscapular Skin Fold, SISF: Supra Iliac Skin Fold, ABSF: Abdominal Skin Fold, TBW: Total Body Water, FFM: Fat-free mass, FM: fat mass, BF%: Percent body fat, BMR: Basal metabolic rate, Chol:: Total cholesterol, TG: Triglyceride, HDL-C : High-density lipoproteins cholesterol, LDL: Low- density lipoprotein- cholesterol

*P value > 0.05, insignificant difference

**Table 2:** Mann-Whitney test showing a comparison between the obese (BMI ≥95th percentile) and the control (BMI>15th - <85th percentiles) groups regarding the investigated clinical, anthropometric, body composition and laboratory parameters

Parameter	Obese (*n* = 72)		Control (*n* = 39)		z	p
	Mean	SD	Mean	SD		
Blood pressure						
SBP (mm Hg)	106.971	15.417	94.871	9.63	5.89	0.000**
DBP (mm Hg)	66.111	10.948	58.718	3.187	5.88	0.000**
Anthropometry						
Weight (Kg)	58.293	11.222	23.59	8.804	8.42	0.000**
Height (cm)	140.65	11.375	120.15	16.701	6.07	0.000**
BMI (kg/m^2^)	29.281	2.546	15.732	1.709	8.68	0.000**
WC (cm)	90.31	9.735	58.13	7.146	8.52	0.000**
HC (cm)	99.6	13.341	65.46	8.873	8.45	0.000**
WHR (cm/cm)	0.9125	0.0761	0.8872	0.0703	2.58	0.010**
WHTR (cm/cm)	0.644	0.064	0.489	0.061	8.11	0.000**
Skin fold thickness						
BSF (mm)	20.64	6.36	7.87	1.82	8.45	0.000**
TSF (mm)	30.29	7.04	11	2.87	8.41	0.000**
SCSF (mm)	34.67	9.69	7.97	4.02	8.49	0.000**
SISF (mm)	30.05	9.73	7.54	3.24	8.43	0.000**
ABSF (mm)	34.71	9.09	7	2.24	8.56	0.000**
Peripheral obesity (mm)	50.93	12.68	18.87	4.57	8.39	0.000**
Central obesity (mm)	99.42	20.96	22.51	9.15	8.54	0.000**
Body composition						
TBW (Kg)	60.52	10.5	12.22	3.38	8.44	0.000**
FFM (Kg)	33.85	5.5	16.74	4.64	8.23	0.000**
FM (Kg)	26.66	6.44	2.86	7.39	8.08	0.000**
BF%	42.13	6.52	7.58	29.41	6.71	0.000**
BMR (Kcal)	1682.65	197.44	3445.62	408.2	-8.14	0.000**
Laboratory parameters						
Lipid profile						
Chol (mg/dl)	175.33	30.64	161.34	29.45	2.17	0.030*
TG (mg/dl)	88.08	28.79	85.05	26.74	0.87	0.387
HDL (mg/dl)	46.18	20.57	54.85	14.4	-3.6	0.000**
LDL (mg/dl)	68.15	9.29	66.14	8.86	0.995	0.320
Zonulin (ng/mL)	1.07	1.62	1.21	1.05	-2.11	0.029*
Copeptin (pmol/l)	222.32	103.47	164.85	112.02	2.16	0.031*

SBP: Systolic blood pressure, DBP: Diastolic blood pressure, BMI: Body Mass Index, WC: Waist circumference, HC: Hip circumference, WHR: Waist/Hip ratio, WHTR: Waist/Height ratio, BSF: Biceps Skin Fold, TSF: Triceps Skin Fold, SCSF: Subscapular Skin Fold, SISF: Supra Iliac Skin Fold, ABSF: Abdominal Skin Fold, TBW: Total Body Water, FFM: Fat-free mass, FM: fat mass, BF%: Percent body fat, BMR: Basal metabolic rate, Chol:: Total cholesterol, TG: Triglyceride, HDL-C : High-density lipoproteins cholesterol, LDL: Low-density lipoprotein- cholesterol

*P value < 0.05, significant

**P value < 0.001, highly significant

**Table 3:** Spearman’s correlation between Zonulin and Copeptin with clinical, anthropometric, body composition and laboratory parameters among the obese children (BMI ≥ 95th percentile)

Parameter	Zonulin (ng/mL)		Copeptin (pmol/l)	
	r	p-value	r	p-value
SBP (mm Hg)	− 0.094	0.439	0.089	0.469
DBP (mm Hg)	− 0.178	0.139	0.193	0.115
Anthropometry				
Weight (Kg)	− 0.214	0.077	0.288	0.017*
Height (cm)	− 0.051	0.678	0.071	0.563
BMI (kg/m^2^)	− 0.400	0.001*	0.488	0.000**
WC (cm)	− 0.325	0.006*	0.378	0.001*
HC (cm)	− 0.482	0.000**	0.504	0.000**
WHR (cm/cm)	− 0.048	0.693	0.001	0.991
WHTR (cm/cm)	− 0.323	0.006*	0.369	0.002*
Skin fold thickness				
BSF (mm)	− 0.148	0.238	0.139	0.274
TSF (mm)	− 0.178	0.155	0.269	0.032*
SCSF (mm)	− 0.287	0.002*	0.338	0.006*
SISF (mm)	− 0.016	0.897	0.028	0.828
ABSF (mm)	− 0.229	0.066	0.351	0.004*
Peripheral obesity (mm)	− 0.162	0.197	0.204	0.106
Central obesity (mm)	− 0.239	0.056	0.336	0.007*
Body composition				
TBW (kg)	− 0.323	0.011*	0.415	0.001*
FFM (Kg)	− 0.359	0.004*	0.441	0.000**
FM (Kg)	− 0.269	0.035*	0.333	0.009*
BF%	− 0.003	0.981	0.051	0.693
BMR (Kcal)	− 0.369	0.006*	0.445	0.001*
Laboratory				
Chol (mg/dl)	− 0.053	0.665	0.147	0.231
TG (mg/dl)	0.215	0.074	− 0.176	0.151
HDL (mg/dl)	0.009	0.938	0.015	0.905
LDL (mg/dl)	− 0.004	0.971	0.035	0.077
Zonulin (ng/mL)			-0.801	0.000**
Copeptin (pmol/l)	− 0.801	0.000**		

SBP: Systolic blood pressure, DBP: Diastolic blood pressure, BMI: Body Mass Index, WC: Waist circumference, HC: Hip circumference, WHR: Waist/Hip ratio, WHTR: Waist/Height ratio, BSF: Biceps Skin Fold, TSF: Triceps Skin Fold, SCSF: Subscapular Skin Fold, SISF: Supra Iliac Skin Fold, ABSF: Abdominal Skin Fold, TBW: Total Body Water, FFM: Fat-free mass, FM: fat mass, BF%: Percent body fat, BMR: Basal metabolic rate, Chol: Total cholesterol, TG: Triglyceride, HDL-C: High-density lipoproteins cholesterol, LDL: Low- density lipoprotein- cholesterol

*P value < 0.05, significant. **P value < 0.001, highly significant

**Table 4:** Spearman’s Correlation between Zonulin and Copeptin with clinical, anthropometric, body composition and laboratory parameters among the control children (BMI > 15th - <85th percentiles)

Parameter	Zonulin (ng/mL)		Copeptin (pmol/l)	
	r	p-value	r	p-value
SBP (mm Hg)	− 0.036	0.828	0.055	0.740
DBP (mm Hg)	0.009	0.959	0.140	0.397
Anthropometry				
Weight (Kg)	− 0.548	0.000**	0.584	0.000**
Height (cm)	− 0.578	0.000**	0.576	0.000**
BMI (kg/m^2^)	− 0.143	0.387	0.154	0.350
WC (cm)	− 0.299	0.064	0.090	0.587
HC (cm)	− 0.346	0.031*	0.425	0.007*
WHR (cm/cm)	0.250	0.125	− 0.338	0.035*
WHTR (cm/cm)	0.510	0.001*	− 0.609	0.000**
Skin fold thickness				
BSF (mm)	0.256	0.116	− 0.456	0.004*
TSF (mm)	0.141	0.394	− 0.377	0.018*
SCSF (mm)	0.174	0.290	− 0.369	0.021*
SISF (mm)	− 0.089	0.592	− 0.118	0.473
ABSF (mm)	0.306	0.058	− 0.450	0.004*
Peripheral obesity (mm)	0.218	0.182	− 0.447	0.004*
Central obesity (mm)	0.109	0.510	− 0.299	0.065
Body composition				
TBW (kg)	0.106	0.523	0.183	0.264
FFM (Kg)	0.152	0.354	0.128	0.436
FM (Kg)	− 0.310	0.055	0.135	0.413
BF%	− 0.242	0.137	0.010	0.950
BMR (Kcal)	0.121	0.464	0.172	0.295
Laboratory				
Chol (mg/dl)	0.019	0.910	0.148	0.368
TG (mg/dl)	− 0.350	0.029*	0.402	0.011*
HDL (mg/dl)	0.398	0.012*	− 0.150	0.361
LDL (mg/dl)	0.263	0.105	− 0.169	0.302
Zonulin (ng/mL)			-0.778	0.000**
Copeptin (pmol/l)	-0.778	0.000**		

SBP: Systolic blood pressure, DBP: Diastolic blood pressure, BMI: Body Mass Index, WC: Waist circumference, HC: Hip circumference, WHR: Waist/Hip ratio, WHTR: Waist/Height ratio, BSF: Biceps Skin Fold, TSF: Triceps Skin Fold, SCSF: Subscapular Skin Fold, SISF: Supra Iliac Skin Fold, ABSF: Abdominal Skin Fold, TBW: Total Body Water, FFM: Fat-free mass, FM: fat mass, BF%: Percent body fat, BMR: Basal metabolic rate, Chol:: Total cholesterol, TG: Triglyceride, HDL-C: High-density lipoproteins cholesterol, LDL: Low- density lipoprotein- cholesterol

*P value < 0.05, significant. **P value < 0.001, highly significant

## Discussion

Obese children are more likely to become obese adults who are more likely to have chronic diseases. Moreover, they may develop early symptoms of chronic disease without being conscious of the problem, and consequently worsen the probable disease complications [21].

Insignificant sex differences were recorded in the current study; particularly in serum Zonulin and Copeptin. This came in agreement with previous studies which revealed insignificant sex difference among obese children and adolescents in the serum Zonulin level [11, 22, 23], and serum Copeptin [2, 24, 25] .

The present study found that obese children had significantly higher values than normal-weight healthy children, in all the studied obesity markers (anthropometric measurements), fat distribution, and body composition parameters; except BMR; and total cholesterol, and significantly lower value of HDL and BMR, but there were insignificant differences between obese and control children regarding LDL and TG.

In agreement with the current results, the increased values of the anthropometric parameters (BMI, WC, HC, WHR and WHTR) and body composition (FM and BF %) in obese children more than the normal weight ones were reported in different studies [11, 23, 26, 27]. Coinciding with current laboratory findings, significantly higher total cholesterol and significantly lower HDL-C were reported by *Tuncay et al.* [7], *Song et al.* [28], *and Martin et al.* [29] in obese children and adolescents.

In contrary to current results, *Tuncay et al.* [7], *Liudmyla et al.* [10] *and Martin et al.* [29], recorded significant higher triglycerides and LDL-C; in obese children and adolescents. While similar HDL-C levels in both obese and normal weight children was recorded by *Yin et al.* [25]. This might be attributed to differences of age group, ethnicity or effect of puberty.

The current results revealed that; the obese group showed significantly higher value of Copeptin and a lower value of Zonulin than the control one. There was significant negative correlation between serum Zonulin and Copeptin among both obese and control children. Among the obese group, levels of serum Zonulin revealed significant negative correlations, while Copeptin showed significant positive correlations with some obesity markers (BMI, WC, HC, WHTR, SCSF) and body composition (TBW, FFM and FM and BMR), and insignificant correlations with blood pressure and lipid profile. In addition, Copeptin had significant positive correlations with fat distribution (TSF, ABSF and central obesity index).

Similar with the results of the current study, higher Copeptin level was reported by Gerdi et al. [2], among obese children than in non-obese ones, and by *Tenderenda-Banasiuk et al.* [30] and *Rothermel et al.* [31] among both obese children and adolescents. Hossam et al. [13] reported significant correlation between serum Copeptin level and BMI. Saleem et al. [32] and *Enhörning et al.* [33]. attributed the elevated serum Copeptin level in overweight children and adolescents to the role played by Copeptin, as one of the mediators associated with chronic stress like obesity and metabolic syndrome, i.e. activation of the hypothalamic-pituitary-adrenal axis by AVP.

In disagreement to current findings, *Schiel et al.* [34] found that serum Copeptin level, in diabetic obese children, was lower than that of the healthy control group. *Lewandowski et al.* [35] also found lower serum Copeptin levels in obese adults compared to normal-weight ones. *Gohar et al.* [36] observed an insignificant association between the levels of serum Copeptin and both body weight and waist circumference “WC”, when adjusting their results for age and BMI. Moreover, a significant higher level of Zonulin level was reported by *Tuncay et al.* [7], *Kim et al.* [37], *Liudmyla et al.* [10]. , *and Strashok et al.* [11], in obese children and adolescents.

Moreover, *Tuncay et al.* [7] found positive correlation between serum Zonulin levels and BMI, BF% and LDL-C., whereas in obese and normal weight/control male groups it was negatively correlated with HDL-C. *Lusikelelwe et al.* [9] found that; among overweight/obese females; Zonulin was positively associated with BMI. This may be explained by the sample sex and age difference; as that of *Lusikelelwe et al.* comprised South African adult females only, but that of the current study included Egyptian children of both sexes.

*Strashok et al.* [11] found positive correlations between Zonulin level, body weight and abdominal fat distribution. *Liudmyla et al.* [10] also found positive correlations between Zonulin level and body weight in adolescents. This may be explained by the different age group of the current study and the effect of puberty.

It is worth mentioning that Zonulin in the current study correlated negatively with the obesity markers (most of the anthropometric measurements) and body composition. It may be related to the fact that nobody has a clear understanding of the physiological significance of the structural differences between the children and adult Zonulin. Hence, these differences could further support the possibility of multiple functions of Zonulin in children [9–11]. *Lusikelelwe et al.* [9] investigated the association between the metabolic imbalance with obesity and intestinal permeability, and suggested that gut permeability could be the cause, the consequence, or both of obesity and its metabolic disorders. In addition, they hypothesized that Zonulin level represents more interstitial injury than inflammation caused by the injury, thus reflecting instead the extent (area) rather than severity of intestinal injury [38]. This could explain the findings of the present study, although this necessitates further studies.

Reviewing the literature, the association between the serum levels of both Zonulin and Copeptin, in children, hasn’t yet been investigated. Nevertheless, few studies have discussed such relations in adult individuals [39, 40].

In summary, among obese children, serum Zonulin and Copeptin had reverse (negative and positive respectively) significant correlations with the obesity markers and body composition parameters; and insignificant correlations with either lipid profile or blood pressure. Moreover serum Copeptin had positive significant correlations with the fat distribution.

## Conclusion

Among Egyptian children, Zonulin and Copeptin had significant negative relation with each other, and Copeptin is related more to obesity than Zonulin. However they cannot be used as predictors of metabolic disturbance, as they had insignificant correlations with lipid profile or blood pressure.

## Data Availability

The datasets used and/or analyzed during the current study are available from the corresponding author on reasonable request, after taking the permission of our institute “National Research Centre”.
